# Two-stage revision for periprosthetic joint infection in unicompartmental knee arthroplasty: clinical and radiological results

**DOI:** 10.1007/s00402-022-04464-8

**Published:** 2022-05-19

**Authors:** Luca Cavagnaro, Francesco Chiarlone, Lorenzo Mosconi, Andrea Zanirato, Matteo Formica, Giorgio Burastero

**Affiliations:** 1 Joint Replacement Unit/Bone Infection Unit, Ospedale Santa Corona, Via XXV Aprile 38, 17027 Pietra Ligure, SV Italy; 2 Orthopaedic Department, Policlinico San Martino Largo, Rosanna Benzi 10, 16132 Genoa, GE Italy; 3 Joint Replacement Unit, Istituto Ortopedico Galeazzi, Via Riccardo Galeazzi 4, 20161 Milan, MI Italy

**Keywords:** Unicompartmental knee arthroplasty, Periprosthetic joint infection, Two-stage revision, Outcomes

## Abstract

**Introduction:**

Unicompartmental knee arthroplasty (UKA) has an infection rate of 0.1–0.8%. Despite the wide amount of literature about septic total knee arthroplasty management, few data are available for UKA infection treatment. The aim is to present the clinical and radiological outcomes along with complication rates of a series of septic UKA treated with two-stage exchange.

**Methods:**

We retrospectively reviewed 16 patient treated with staged UKA revision for infection between June 2015 and September 2019 in a single bone infection unit. The main demographic and surgical data were recorded. Clinical scores (VAS, KSS, OKS, postoperative ROM), radiological parameters (osseointegration, loosening and radiolucencies) and complications were reported. The mean follow-up was 33.5 ± 6.9 months.

**Results:**

Mean age at surgery was 68.5 ± 9.1. All but two were medial UKA. The mean number of previous surgeries was 2.9 ± 1.9. The mean ROM, VAS, KSS and OKS of the entire population improved significantly (p < 0.01). Radiological analysis did not show any migration or implant loosening. Ten constrained condylar and six posterior stabilized prosthesis were finally implanted. One intraoperative pathogen isolation was recorded and managed with suppressive therapy and good final outcome. The implant survivorship free from infection was 100% at the final follow-up. The overall survival rate for any reason of revision was 100%

**Conclusion:**

According to our results, staged revision represents a reliable ad effective option in delayed and late UKA infections. This technique provides optimal clinical and radiological results with acceptable complication rates. To the best of our knowledge, this represent the widest case series on infected UKA managed with two-stage exchange.

## Introduction

Unicompartmental knee arthroplasty (UKA) is an increasingly popular surgical procedure that with proper indications leads to a significant improvement in knee function and patient satisfaction providing long-term survival rates that exceed 90% [1, 2]. According to national joint replacement registries, UKA usage is reported at 2–12% in clinical practice [3–6].

Between 1998 and 2005, the UKAs performed in the USA increased almost eightfold [7].

Pperiprosthetic joint infection (PJI) is a well-known complication after total knee arthroplasty (TKA), occurring in 1–2% of cases. In UKA, a lower rate of PJI was reported (0.1–0.8%). Nevertheless, according to the increasing number of UKAs implantation, also management of PJI will be a growing clinical problem. The Society of Unicondylar Research and Continuing Educations confirmed the validity of laboratory tests and corresponding cutoff values in the diagnosis of PJI after UKA [8]. Nevertheless, the guidelines for the management of PJI after UKA are limited; the debridement and implant retention (DAIR) approach was condoned in both acute and chronic situations based on limited evidence [9, 10].

In literature, previous studies reported results of a mixed populations of acute and chronic PJI after UKA managed with different approaches (DAIR, two-stage or one-stage exchange) [9, 11, 12].

In this context, the aim of this observational study is to report and analyze the clinical and radiological results, long-term survivorship and complication rate of a series of septic UKAs managed with two-stage exchange.

## Materials and methods

All data had been prospectively collected by our Institutional Arthroplasty Registry from June 2015 to September 2019 and then analyzed. The institutional review board (IRB) approved this single-center study. Written and informed consent was obtained from all the included participants. All procedures were conducted according to the Declaration of Helsinki. The Strengthening the Reporting of Observational Studies in Epidemiology (STROBE) guidelines were used for manuscript drafting.

All patients undergoing a two-stage knee revision for septic UKA treatment, and with a minimum follow-up of 24 months, were enrolled in this study. Patients who underwent knee revision surgery with other techniques were excluded.

Periprosthetic joint infection (PJI) diagnosis was made according to the modified Musculoskeletal Infection Society (MSIS) criteria [13, 14]. The Zimmerli classification was used for PJI classification [15].

Femoral and tibial bone defects were classified radiographically in the preoperative stage and confirmed during surgery according to the Anderson Orthopaedic Research Institute (AORI) classification [16].

The main demographic (age, sex, diagnosis, affected side, medial or lateral UKA, body mass index (BMI), comorbidities, previous surgical procedures, American Society of Anesthesiology (ASA) score, McPherson stage) surgical (inter-stage period, complication during the inter-stage, surgical time at first and second stage, surgical approach and final component implantation) and microbiological data were recorded.

### Clinical and radiographic evaluation

Clinical and radiographic evaluation were performed before the first and the second stage and after reimplantation at 45 days, 3, 6 and 12 months, and annually thereafter. Clinical assessment included physical examination, the visual analog scale (VAS) score, the Knee Society Score (KSS), the Oxford Knee Score (OKS), the passive and active range of movement (ROM) along with flexion contracture of extension lag. The ROM was determined with the use of a standard clinical goniometer. Standing AP, lateral, long-leg and Merchant radiographs were performed in the preoperative and at 45 days follow-up. Standing AP, lateral, and Merchant plain X-ray analysis were performed during the other follow-up time points.

Radiological evaluation was carried out according to the Knee Society total knee arthroplasty radiographic evaluation [17]. The scoring system for long-stemmed revision prostheses was adopted if needed [18] to fully evaluate the entire length of the prostheses. Radiographs were assessed by two orthopedic fellows specifically trained on knee reconstructive surgery. Doubtful cases were solved by consensus. Osseointegration, migration, loosening, osteolysis and malposition were evaluated. Implant axial alignment was evaluated with neutral defined as between 3° and 9° of valgus [19].

Every possible complication (wound drainage, deep infection, aseptic loosening, intraoperative or postoperative fractures, revision, reoperation) related to the operated knee was recorded.

The authors considered as revision any kind of surgical procedure after the indexed operation that required fixed component removal. Reoperation was defined as any kind of surgery that involved the specific knee joint after the indexed procedure with or without implant component removal. We defined persistent infection as each PJI after the second stage or positive culture at reimplantation with isolation of the original infecting organism [20]. A new infection was defined according to the MSIS criteria [13, 14].

### Surgical procedure

All two-stage TKA revision procedures were performed by a skilled surgeon experienced in complex revision arthroplasty. A standard medial parapatellar approach was used in all knees. During explantation, after a deep surgical debridement, a mobile antibiotic-loaded spacer was implanted. A handmade stem was added as reinforcement if needed. A course of 14 days of intravenous antibiotic therapy or longer was always performed. The switch to a specific antibiotic oral therapy was performed according to microbiological results. Patients with negative culture were switched to empirical oral antibiotic therapy. Sonication of the infected implant, one specimen for definitive histology and three to six intraoperative biopsies for microbiological analysis were routinely obtained from the area macroscopically most suspicious of infection. Histopathological examination of the periprosthetic membrane was performed using the classification established by Krenn and Morawietz [21]. In the laboratory, retrieved implants were immersed in solution and treated in an ultrasonic bath for 60 s at 80% *P* = 160 W. Subsequently, 10 ml of sonicate fluid was placed in aerobic and anaerobic blood culture bottles and cultured. The cutoff value for sonication was 5 CFU/ml fluid.

All patients underwent a staged algorithm of 6 weeks of antibiotic therapy (2 weeks of intravenous antibiotic therapy, followed by 4 weeks of oral administration if possible) and 2 weeks of washout. Concurrent medical comorbidities delayed the second stage in three patients. Another patient was reimplanted at 6 months after the first stage because of a concomitant UNI-PJI and a supracondylar fracture.

During reimplantation and after spacer removal, a new surgical debridement was performed. Three to six intraoperative samples were taken for microbiological analysis, as well as one specimen for frozen section and definitive histology. Prosthetic design and constraint choice were defined during surgery according to the intraoperative situation.

### Postoperative course

Partial weight-bearing with crouches started on the second postoperative day after removal of the surgical drain. Full weight-bearing was allowed after 6 weeks from surgery, whenever possible. Passive and progressive knee mobilization started on the first day after surgery and continued for the first 6 weeks. One patient with simultaneous lateral UKA infection and supracondylar femoral fracture was managed with stemmed spacer implantation and postoperative cast for 45 days. Standard venous thromboembolism prophylaxis with enoxaparin and compression stockings was prescribed at least for 45 days. In agreement with the infectious disease team, after the second stage a specific intravenous antibiotic course was administered until intraoperative microbiological results were attained and continued thereafter if necessary.

### Statistical analysis

Continuous variables were reported as mean ± standard deviation (SD) and compared between preoperative and final follow-up using the Student’s *t* test. Categorical variables were expressed as the number of cases or percentage. For all the analyzed data, a two-tailed, *p *value < 0.05 was considered statistically significant. Inter-observer reliability was evaluated with the Cohen’s kappa coefficient. Kaplan–Meier survival curves with 95% confidence intervals (CI) were created to analyze final implant survivorship free of revision for any reason as the end points.

## Results

### Demographic data

Sixteen patients undergoing staged knee revision for UKA PJI were included in the current study. All PJI were classified as delayed (chronic PJI > 3 months after implantation) or late (chronic low-grade PJI > 24 months after implantation) infections according to Zimmerli classification. No acute hematogenous infections were included in the study. The mean age was 68.5 ± 9.1 years. Nine patients were men (56.2%) and seven were women (42.8%). The average BMI was 27.8 ± 3.9 kg/m^2^. The median follow-up was 33.5 ± 6.9 months (range 25–57 months). No patient was lost during the follow-up. The mean number of previous surgical procedures was 2.9 ± 1.8, excluding the indexed two-staged revision. Fourteen patients had a medial UKA infection, and a lateral UKA was revised in two patients. One patient with lateral UKA had a concurrent supracondylar femoral fracture. The main demographic data are reported in Table 1.

**Table 1:** Demographic data

**Parameter**	
Gender	
Male	9 (56.2)
Female	7 (43.8)
BMI [kg/m^2^] (body mass index)*	27.8 ± 3.9
Age at time of surgery [years]*	68.5 ± 9.1
Laterality	
Right	9 (56.2)
Left	7 (43.8)
Revision diagnosis	
2-stage reimplantation for UKA PJI	16 (100)
UKA side	
Medial	14 (87.5)
Lateral	2 (12.5)
Number of previous surgeries*	2.9 ± 1.8
Smoking status	
Current	4 (25.0)
Former	3 (18.8)
Never	9 (56.2)
ASA score	
ASA 2	4 (25.0)
ASA 3	10 (62.5)
ASA 4	2 (12.5)
Type of infection	
Delayed (chronic PJI > 3 months after implantation)	3 (18.8)
Late (chronic low-grade PJI > 24 months after implantation)	13 (81.2)
McPherson staging system	
III A 1	1 (6.2)
III A 2	3 (18.8)
III B 1	5 (31.3)
III B 2	4 (25.0)
III B 3	2 (12.5)
III C 2	1 (6.2)
Comorbidity	
Diabetes	9 (56.2)
Cardiopathy	3 (18.8)
Substance abuse	1 (6.2)
Renal failure	1 (6.2)
Hepatopathy	1 (6.2)

Presented as *n* (%), except * presented as mean ± standard deviation

*BMI* body mass index, *UKA* unicompartmental knee arthroplasty, *PJI* periprosthetic joint Infection, *ASA* American Society of Anesthesiology

The indication for TKA revision was delayed or late UKA PJI (> 3 months from surgery) in all of the included patients (100%). Microbiological analysis revealed three coagulase-negative staphylococci (CoNS), three Gram-negative, one methicillin-resistant *Staphylococcus aureus* (MRSA), two methicillin-sensitive *Staphylococcus aureus* (MSSA), one *Enterococcus faecalis* culture and three polymicrobial infections (specifically, 1 patient with methicillin-sensitive *Staphylococcus epidermidis* and *Staphylococcus capitis*, 1 patient with MRSA and *Klebsiella Pneumoniae* and 1 patient with methicillin-resistant *Staphylococcus epidermidis* and *Micrococcus luteus*). No pathogen isolation was observed in three patients and infection was confirmed according to MSIS criteria. Microbiogical data are summarized in Table 2.

**Table 2:** Microbiology of infected UKA

Microbiology	
Positive culture	13
Methicillin-resistant *S. aureus*	1
Methicillin-sensitive *S. aureus*	2
Polymicrobial flora	3
Coagulase-negative *staphylococci*	3
Gram negative	3
*Enterococcus faecalis*	1
Negative culture	3

*UKA* unicompartmental knee arthroplasty

On the femoral side, ten AORI 1, three AORI 2A, two AORI 2B and one AORI 3 were recorded. Three AORI 1, seven AORI 2A and six AORI 2B tibial bone defects were observed. Mean inter-stage period was 15.3 ± 10.5 weeks. Mean surgical times at the first and second stage were 108.4 ± 30.6 and 126.7 ± 35.0, respectively. No inter-stage complication was observed.

A condylar constrained knee arthroplasty (Nexgen LCCK, Zimmer-Biomet, Warsaw, IN) was implanted in ten cases (62.5%) and a posterior-stabilized knee prosthesis (Nexgen PS, Zimmer-Biomet, Warsaw, IN) in six (37.5%). In all cases of condylar constrained prosthesis implantation, a hybrid fixation and uncemented stems were used. Ten porous tantalum cones (Zimmer-Biomet, Warsaw, IN) were implanted in seven patients (43.8%) for bone defect management. The mean number of augments was 1.7 ± 1.0; the majority of those were used on the medial side of the tibia. Surgical data are reported in Table 3.

**Table 3:** Surgical and implant-related data

Parameter		
AORI defect	Femur	Tibia
1	10 (62.5)	3 (18.6)
2A	3 (18.6)	7 (43.8)
2B	2 (12.5)	6 (37.5)
3	1 (6.2)	
Level of constraint		
PS	6 (37.5)	
CC	10 (62.5)	
Number of cones *	10	
Mean number of cones**	0.8 ± 0.8	
Side of cones		
Femoral	0 (0)	
Tibial	4 (25.0)	
Both	3 (18.6)	
Number of augments *	15	
Mean number of augments **	1.7 ± 1.0	
Side of augments		
Femoral	9 (56.3)	
Tibial	6 (37.5)	
Number of stems *	22	
Stem side		
Femoral	0 (0.0)	
Tibial	2 (12.5)	
Both	10 (62.5)	
Lenght of stems	Femur	Tibia
30	0 (0.0)	2 (12.5)
75	8 (50.0)	9 (56.3)
100	2 (12.5)	1 (6.2)
Mean polyethylene thickness**	11.7 ± 1.6	

Presented as *n* (% relatively to the number of include patients), except * presented as n and ** presented as mean ± standard deviation

*AORI* Anderson Orthopaedic Research Institute, *PS* posterior stabilized, *CC* constrained condylar

### Clinical evaluation

The mean KSS and OKS of the patients who underwent two-stage revision for UKA infection improved significantly from 44.4 ± 11.6 and 19.9 ± 4.6 preoperatively to 84.7 ± 6.7 and 39.2 ± 5.7, respectively, at the last follow-up. (*p* < 0.01). Mean VAS score decreased from 8.1 ± 2.2 to 1.7 ± 1.9 at the last evaluation (*p* < 0.01).

The ROM improved from 56.2 ± 19.4 of mean preoperative flexion to 98.1 ± 12.8 degrees of postoperative flexion at the final follow-up (*p* < 0.01). Mean preoperative flexion contracture was 5.2 ± 6.9 with three patients suffering from flexion contracture more than 10 degrees. Full extension was achieved in all the included patients. No extension lag was recorded at the final follow-up. One patient walked with crutches at the final follow-up for persistent knee pain. Clinical outcomes are summarized in Table 4.

**Table 4:** Clinical outcomes

Score	Preop values	Last f.u	Improvements
	Mean ± SD	Mean ± SD	(*P* value)
KSS	44.4 ± 11.6	84.7 ± 6.7	40.3 (*p* < 0.01)
OKS	19.9 ± 4.6	39.2 ± 5.7	19,3 (*p* < 0.01)
VAS	8.1 ± 2.2	1.7 ± 1.9	6.4 (*p* < 0.01)
Flexion	56.2 ± 19.4	98.1 ± 12.8	41.9 (*p* < 0.01)
Flexion contracture	5.2 ± 6.9	0.0 ± 0.0	5.2 (*p* < 0.01)

*KSS* Knee Score Society, *OKS* Oxford Knee Score, *VAS* visual analog scale

### Radiographic evaluation

**Fig. 1:**
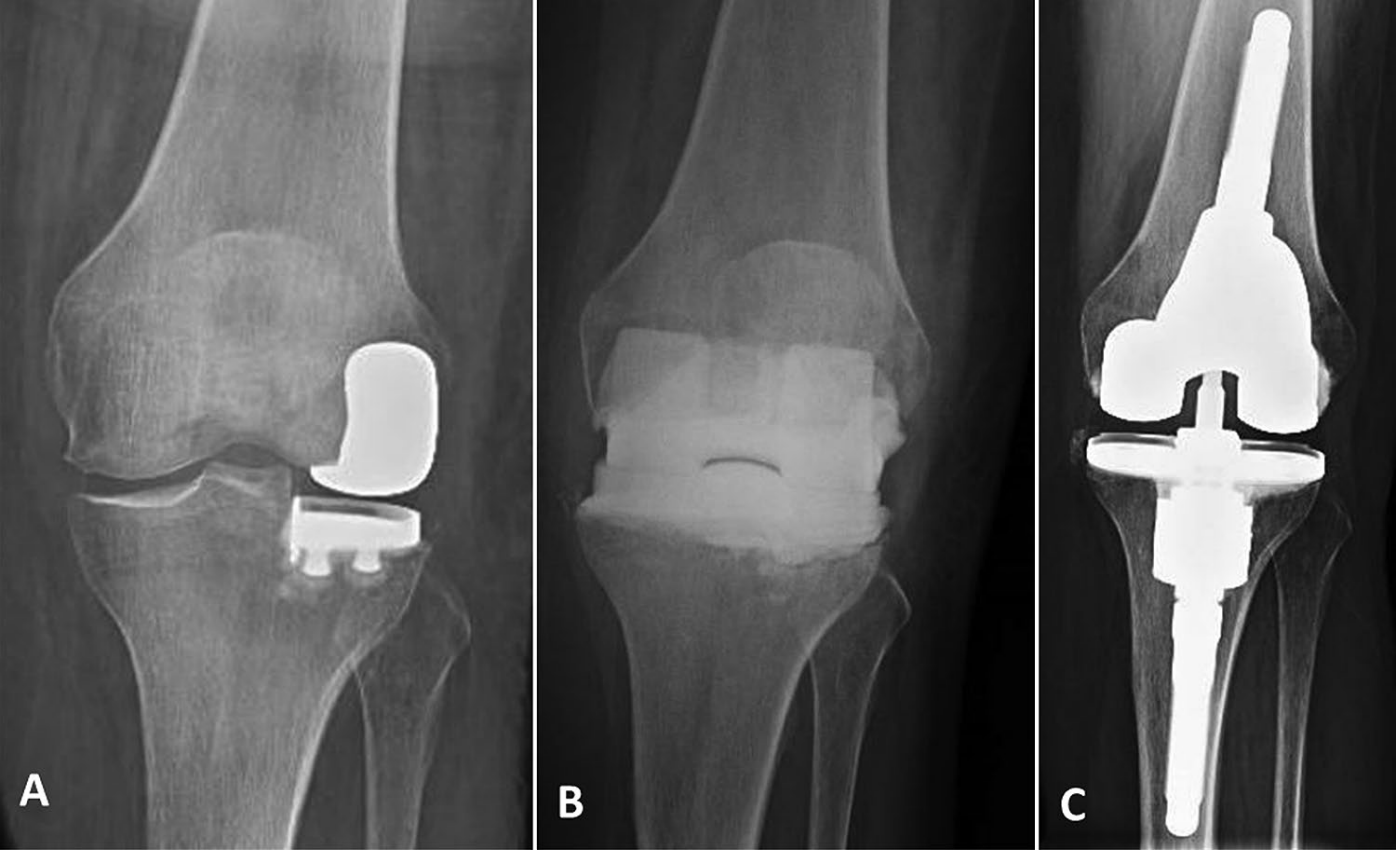
Radiological analysis of a patient affected by left UKA PJI. **A** Preoperative X-ray showing lateral left UKA. **B** Articulating spacer. **C** 3-year follow-up with optimal implant alignment and firm osseointegration of the revision prosthesis

**Fig. 2:**
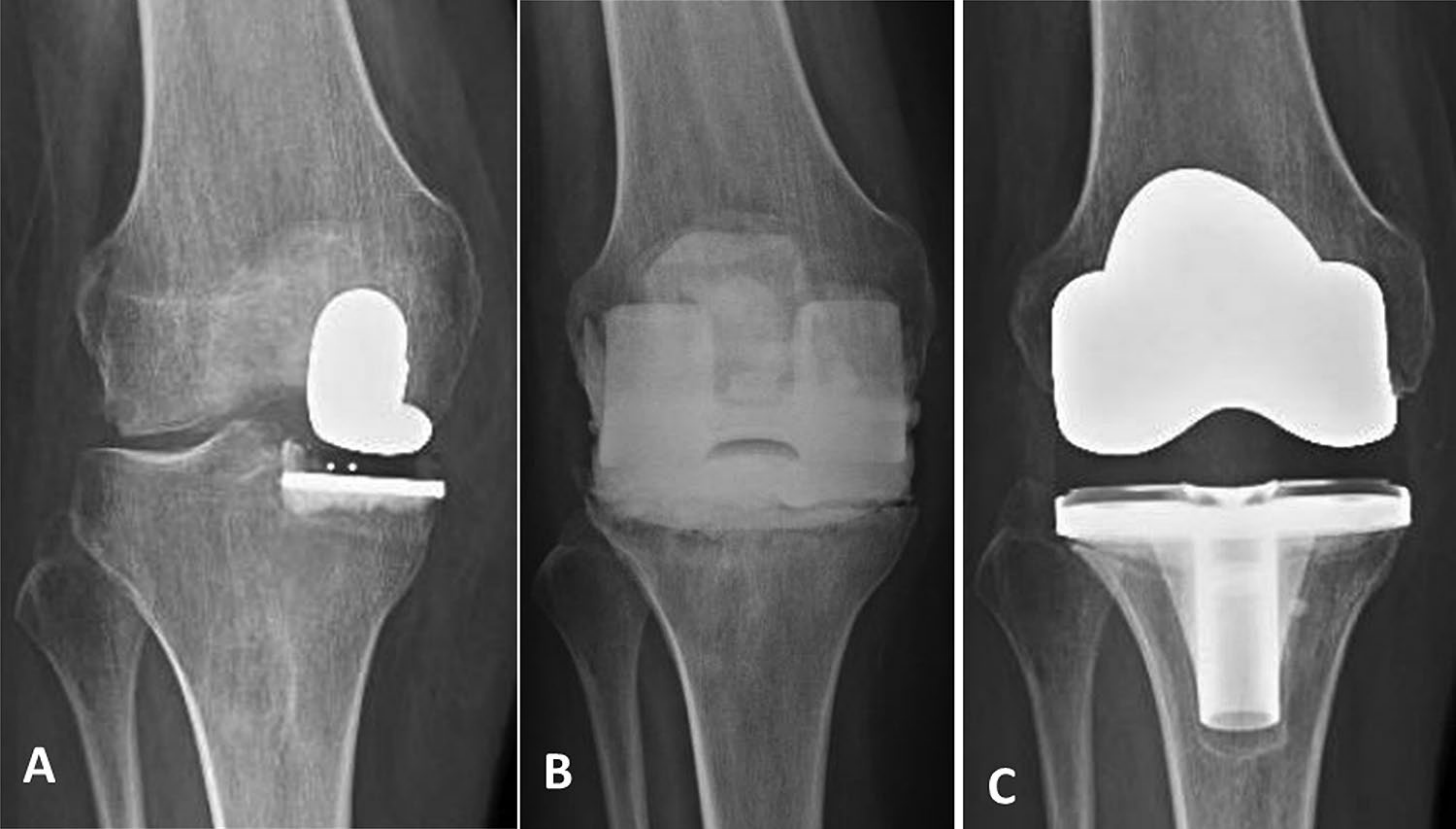
Radiological imaging of a UKA-infected patient. **A** Preoperative X-ray showing a right medial UKA. **B** Articulating spacer. **C** 4-year follow-up with a PS prosthesis implantation

All implants appeared well osseointegrated and well positioned in the AP and LL radiographs (Figs. 1 and 2). No evidence of component loosening or migration was reported at the latest follow-up evaluation.

Radiograph analysis showed all the knee prosthesis implants in neutral position.

Until now, we have reported two (18%) cases of radiolucent lines of less than 1 mm, non-progressive, on the tibial side (zone 1 on the LL view). No cases of femoral or tibial diaphyseal cortical hypertrophy were registered. For all the included radiological parameters, very good (≥ 90%) Cohen's kappa inter-rater agreement was found.

### Complications

During inter-stage interval, no relevant complications were reported.

One (6.25%) of the 16 patients reported a positive culture from spacer sonication.

The positive spacer sonication for *Staphylococcus capitis* was managed with specific suppressive antibiotic therapy for 3 months with excellent final outcome and did not require any revision surgery.

No intraoperative or postoperative fractures occurred. No cases of aseptic loosening were reported. No reoperation or revision was performed after the second-stage procedure. The implant survivorship free from infection was 100% at the final follow-up. Kaplan–Meier analysis showed an overall survival rate of 100% for any reason of revision.

## Discussion

UKA is a reliable procedure in the treatment of single-compartment osteoarthritis with an excellent long-term survivorship and patient-reported outcomes. Although UKA has been reported to have a higher all-cause revision rate when compared to traditional TKA, the number of UKA implanted are increased due to faster recovery, lower morbidity, mortality, rate of periprosthetic joint infection and complications, as well as better patient-reported and functional outcomes than TKA [1, 22–24].

Although it is expected that the number of PJI cases will increase [25, 26], there is currently a paucity of literature surrounding this clinical scenario and no consensus exists regarding the diagnosis and management of UKA PJI. The pathogenesis of PJI in patients with UKAs involves a simultaneous occurrence of implant-related infection and septic arthritis of the native knee. Cartilage damage starts to occur as early as 8 h after infection. Due to infection progression, intra-articular pressure rises with compression and thrombosis of synovial vasculature and further destruction of articular cartilage and soft tissues [27]. The goals of surgical treatment in bacterial septic arthritis of the adult native knee joint include decompression, lavage, debridement, and in some cases synovectomy. [27]. The destruction of native cartilage and instability due to cruciate ligaments damage could determine subsequent arthritis of the contralateral compartment and consequently UKA failure in spite of infections control.

Considering the central role of native cartilage in UKAs PJI, the cartilage could represent a potential cause of high rates of failure after DAIR and a thorough debridement of articular cartilage is crucial for successful infection eradication [11]. This data has been recently confirmed by Russo et al. showing optimal survival rate of native septic arthritis treated by a two-stage procedure [28]. However, this point appears conflictual; it is unclear if debridement of articular cartilage is necessary because treatment of native septic arthritis has been reported to be equally successful with arthroscopic irrigation alone [29].

Especially in chronic situations, a well-known mechanism of disease recurrence is internalization of bacteria (i.e., *Staphylococcus aureus*) by osteocytes and chondrocytes [30]. This feature can explain the rapid infection recurrence even with extensive antibiotic administration and accurate articular lavage.

A clear guideline for the management of PJI after UKA does not exist and is limited to the 2018 International Consensus Meeting (ICM) on PJI [10]. In literature, several approaches were proposed, variously associated with specific antibiotic treatment: DAIR, one-stage and two-stage revision procedures. The overall options satisfy the goals previously reported for bacterial septic arthritis management [27].

The literature suggests that the factors influencing decision regarding PJI treatment after UKAs are: timing of symptoms, type of infection (acute vs chronic), organism, patient comorbidities and local extremity grade. Hernandez et al. suggest that patients who have a longer duration of PJI or have more severe host and extremity status received staged exchange and those who have a shorter duration of PJI received DAIR [11]. Labruyere et al. [12] state that in chronic PJI UKAs factors supporting the decision to perform one-step conversion to TKA are: preoperative identification of the causative organism in the joint aspirate, the susceptibility of this organism to antibiotics, and the feasibility of complete excision.

Analyzing articles concerning UKAs failures, a high success of infection eradication (up to 100%) was reported with exclusion of DAIR procedures (33–50% of infection recurrence) [9, 11].

In chronic PJI, the global survivorship free from septic reoperation was 66.6% and a survivorship from all-cause reoperation was 55.6%. DAIR without chronic suppressive antibiotic therapy demonstrated 100% of failure. The association with chronic suppressive therapy, instead, guarantees survivorship free from septic reoperation of 75%. As demonstrated, DAIR with chronic suppressive antibiotic therapy is not a curative option and should be avoided in chronic PJI. One- and two-stage procedures demonstrated good performance in infection eradication. In particular, Hernandez et al. [11] showed a 100% survival rate at 5 years when initial treatment was two-stage exchange. The presented data compare favorably with results provided by the most recent literature. The authors reported only one patient with positive spacer sonication. This patient has positive preoperative laboratory test and was considered a doubtful case. All tissue samples were negative and histology showed a type IV membrane. Frozen section reported a value of > 5 < 10 polymorphonuclear leukocyte number per high-power field. *Staphylococcus capitis* was finally isolated and managed with a 3-month course of oral antibiotic therapy according to the infectious disease team to get good final results.

Converting a UKA to a TKA may be challenging due to issues of bone loss, need for augmentation, and restoring joint line and rotation.

In both septic and aseptic conversion, the final components are often characterized by hinged or constrained implants with stems, different kinds of augmentation and metaphyseal porous metal devices, such as sleeves and cones [31, 32]. Kahn et al. [33] reported that 26% of patients required bone grafting, while 26% required some form of augmentation, with the commonest site being tibia and the commonest augment being tibial stem. Only 8% of the cohort required revision knee implants. The present paper shows slightly higher rates of augments and revision implants. Indeed, ten (62.5%) of the included patients were finally implanted with a semi-constrained prosthesis. This could be due to the fact that the infection eradication often requires a radical debridement with sacrifice of bone and soft tissue mainly dedicated to knee joint stability. Moreover, no bone graft was used because in septic revision cases, such approach is associated with high risk of late resorption, reinfection and suboptimal final outcome [34].

To our knowledge, this is the first paper that analyzes results obtained from a homogenous cohort of septic UKA treated with a two-stage exchange. According to the available literature [35], the present case series is the most relevant one dealing with staged revision of UKA PJI.

Undoubtedly, this study has several limitations. The retrospective nature of the analysis contains inherent limitations which must be considered when evaluating the results. Although we applied our institutional two-stage surgery protocol to all the patients included, the type of spacer as well as the antibiotic therapy is individualized and this could be a bias into the analysis. The absence of control groups made any considerations on different treatment options not possible, and the small sample size limits statistical power of this analysis. However, the prospective collection of data, the relatively long follow-up, the fact that all the patients underwent a standardized protocol of treatment and follow-up, and diagnosis and surgeries were performed in a standard manner by the same surgeon can be considered strengths of this study.

## Conclusion

Two-stage exchange is a reliable and effective procedure in delayed and late UKA infection. It provides excellent ad long-lasting clinical and radiological results with low complication rate. Further high-quality log-term studies will better clarify the results of different approaches to PJI in UKAs.
